# Yield and quality performance of onion (*Allium cepa* L.) hybrid varieties in response to nitrogen fertilization in Northwest Ethiopia

**DOI:** 10.1186/s12870-025-07959-9

**Published:** 2025-12-16

**Authors:** Yezihalem Getaneh, Tadele Yeshiwas, Daniel Asnake, Selamawit Zelalem

**Affiliations:** 1 Department of Horticulture, Merawi Technical & Vocational Education Training College, Amhara, Ethiopia; 2 Department of Horticulture, College of Agriculture and Environmental Sciences, Bahir Dar University, P.O. Box. 5501, Bahir Dar, Ethiopia

**Keywords:** Fertilization, Growth, Hybrid variety, Nitrogen, Onion, Quality

## Abstract

Onion is one of the most important vegetable crops in Ethiopia, including in the Koga Irrigation Scheme. However, the limited availability of hybrid varieties and the inappropriate application of nitrogen fertilizer are major constraints to onion production. Therefore, a field experiment was conducted to evaluate the effects of nitrogen fertilizer on the growth, yield, and quality of hybrid onion varieties and to determine the optimum nitrogen fertilizer rate while identifying productive hybrid cultivars for economical onion production. The study consisted of four hybrid onion varieties (Red Coach, Russet, Jambar, and Bombay Red) and four levels of nitrogen fertilizer (0, 41, 82, and 123 kg ha⁻¹). The results indicated that the main effects of varieties and nitrogen fertilizer significantly influenced all the tested parameters of onion. The highest marketable bulb yield (57.84 t ha⁻¹) was obtained from the Russet variety with 82 kg ha⁻¹ of nitrogen fertilizer. Based on the partial budget analysis, the highest net benefits (12,419.87 USD ha⁻¹) and the highest marginal rate of return (3061.09%) were recorded from the treatment combination of the Russet variety with 82 kg ha⁻¹ of nitrogen fertilizer. Therefore, growing the Russet variety with 82 kg ha⁻¹ of nitrogen fertilizer is recommended for the economical production of onion in the study area and other areas with similar agro-ecological conditions.

## Introduction

 Onions (*Allium cepa* L.), valued for their culinary versatility and health benefits, belong to the Alliaceae family and originate from the rich soils of Southwest Asia and the Mediterranean. With approximately 750 species in the *Allium* genus—including essential staples like leeks and garlic—onions are a vital vegetable crop worldwide [1]. Onions are widely used to flavor and season diverse dishes and are also valued in many communities for their medicinal properties, including potential roles in managing high blood pressure and lowering the risk of cancer, cardiovascular diseases, and diabetes [2]. Nutritionally, they are low-calorie, nutrient-dense vegetables composed mainly of water (about 89–91%) and carbohydrates (9–10%), including simple sugars and dietary fiber, with only small amounts of protein and negligible fat. They are good sources of vitamins such as C, B6, folate, and biotin, and provide important minerals like calcium and chromium, making them a valuable component of a balanced diet [3, 4]. In addition, onion production contributes significantly to smallholder farmers’ income, enhances household food security, and generates employment opportunities [5]. In Ethiopia, particularly within the Amhara region’s Koga Irrigation Scheme, the potential for onion cultivation is high [6]; however, it is hindered by various challenges such as inadequate agronomic practices, limited access to improved seed varieties, pest and disease pressures, and insufficient extension services [7].

Despite the introduction of hybrid onion varieties by commercial suppliers, their actual yield performance in Ethiopia remains largely uncharted. In the context of improving onion production in northwest Amhara Ethiopia, various hybrid varieties such as *Red Coach*,* Russet*,* Jambar*,* and Bombay Red* have been introduced into the local farming system. These varieties were selected due to their high yield potential and better storability compared to open pollinated varieties [8].

Besides to improved varieties, Nitrogen (N) is essential for onion growth, promoting leaf development, photosynthetic efficiency, and bulb yield [9]. Onions require substantial N and respond well to supplemental fertilization, but optimal application rates depend on crop variety, soil fertility, environmental conditions, and management practices [10]. Current Ethiopian fertilizer recommendations of 82 kg N ha⁻¹ and 40.1 kg P ha⁻¹ adopt a blanket approach [11], which fails to account for the unique conditions of different locations, soil nutrient statuses, and specific crop varieties. Intensified N management can enhance bulb yield and quality [12]; however, excessive N reduces storage life, delays maturity, and promotes bolting, compromising profitability [13]. Conversely, insufficient N limits chlorophyll synthesis, reducing yields [14]. Among primary (N, P, K) and secondary nutrients, N requires standardization due to its high soil mobility, significant depletion under intensive cropping, and critical influence on onion yield and quality, necessitating tailored recommendations for Ethiopian conditions. To improve onion production and productivity, location-specific, agro-ecological, soil nutrient-based, variety-specific, and season-specific evaluations and recommendations are necessary. Given that hybrid varieties are relatively new to the region, their precise N fertilizer requirements remain undetermined.

This study is based on the hypothesis that hybrid onion varieties differ in their growth, yield, and quality responses to N fertilizer rates, and there exists an economically optimal N rate for each variety that maximizes marketable yield and quality without incurring unnecessary input costs or compromising bulb storability. Therefore, this study was conducted to address this gap by determining the effects of N fertilizer on the growth, bulb yield, and quality of hybrid onion varieties and identifying the optimum N fertilizer for the economical production of hybrid onion varieties at Koga Irrigation Scheme. This will empower local farmers, enhance economic benefits, and contribute to the broader agricultural development of Ethiopia. The findings of this research are expected to have broader implications beyond the experimental site by providing evidence-based, variety-specific N recommendations, and supporting more efficient use of N fertilizer, thereby reducing production costs and improving profit margins for smallholder farmers.

This paper is structured as follows. The Introduction section describe the background and justification of the research. The Materials and Methods section describe the experimental design, including N fertilizer treatments, onion varieties, and data collection methods for yield and quality parameters in the Koga Irrigation Scheme. The results section presents the effects of N rates on growth, bulb yield, and quality across tested varieties. The discussion section interprets these findings in the context of Ethiopian agro-ecologies, comparing them with existing literature and addressing implications for farmers. Finally, the conclusion summarizes key findings and provides recommendations for tailored N management in onion production.

## Materials and methods

### Description of the study area

**Fig. 1:**
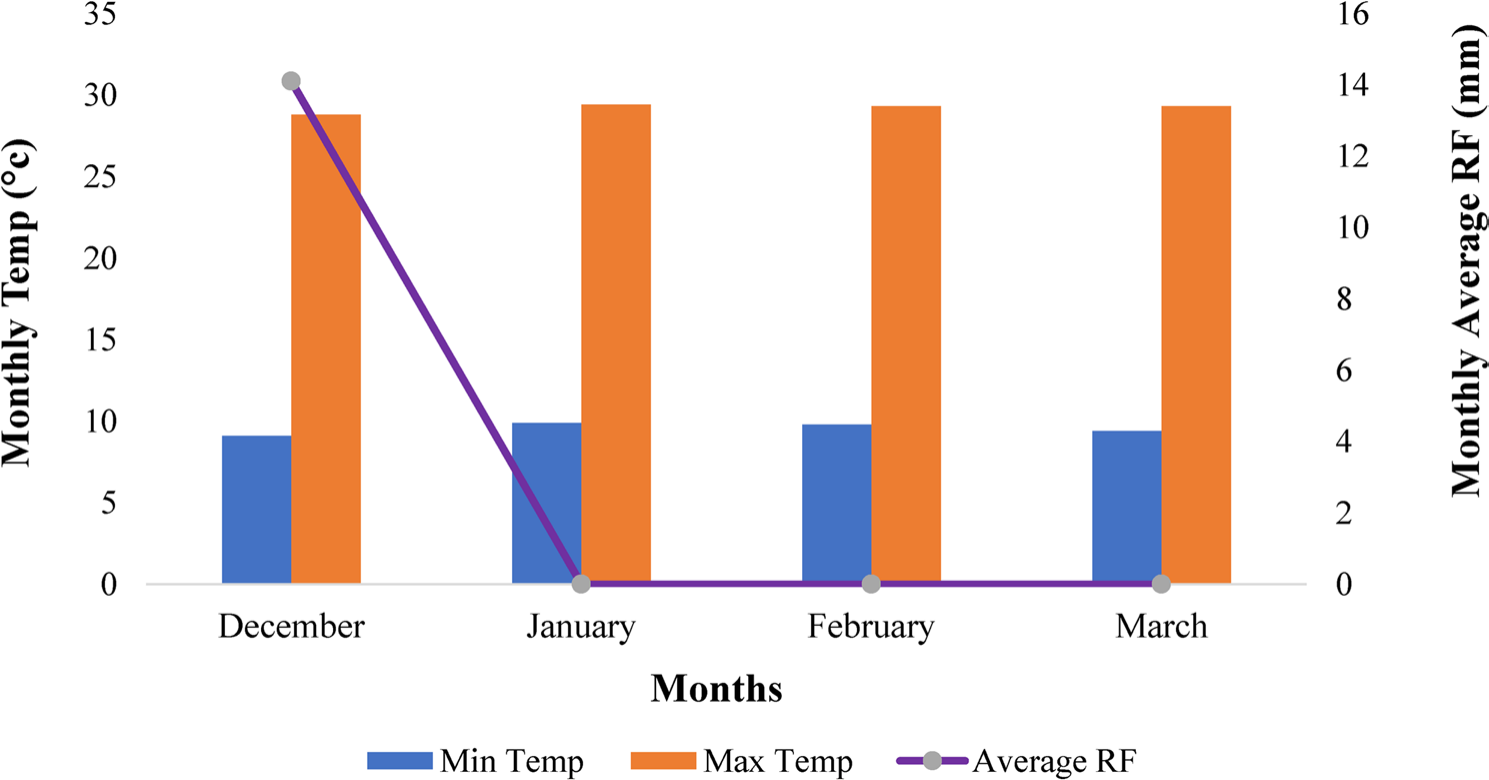
The environmental conditions of the experimental site during the cropping period. Where: Temp = temperature; Min = minimum; Max = maximum; RF = rainfall

The experiment was carried out at the Koga Irrigation Scheme, located in North Mecha Woreda of the West Gojjam Zone, during the 2023/24 off-season (December to March) under irrigated conditions. The area is 37 km far from the capital city of Amhara region, Bahir Dar. The Koga irrigation site lies at 11º24’62” N latitude and 37º08’97” E longitudes with an altitude of 1960 m above sea level [15]. The minimum and maximum temperatures, and rainfall of the Koga experimental site are presented in Fig. 1 [16].

Soil samples from the experimental site were collected via standard procedures prior to planting. A composite soil sample was taken before the experimental plot was plowed by mixing samples collected at the experimental site along the two diagonal lines of the field at 0–20 cm depth via an auger. The physical and chemical properties of the composite soils were analyzed at the Soil and Plant Nutrition Laboratory of the College of Agricultural and Environmental Sciences, Bahir Dar University. The results of the soil analysis are presented in Table 1.

**Table 1:** Status of nutrients in the experimental location before planting in 2023/24 irrigation season

Soil properties	Soil Sample	Soil type
Texture		
Sand (%)	56	Nitisol
Silt (%)	16	
Clay (%)	28	
Textural class	Sandy Clay Loam	
pH_H_2_O (1:2.5)	5.40	
EC (µS/cm)	161.30	
Organic Carbon (%)	2.18	
Organic Matter (%)	3.77	
CEC (cmol (+) kg^− 1^)	27.60	
Total Nitrogen (%)	0.42	
Available Phosphorus (mg/kg) [Olsen method]	10.02	

Where: EC, electrical conductivity; CEC, cation exchange capacity

### Experimental materials

Bombay red, Red coach, Russet and Jambar onion varieties were used as the test crop. Bombay red is one of the most commonly and widely used improved onion variety in Koga Irrigation Scheme. While, Red coach, Russet and Jambar are hybrid onion varieties which are newly introduced cultivars by commercial suppliers. The release year, altitude, and maturity period of each variety are presented in Table 2 [17, 18]. The planting materials were procured from agricultural input suppliers in Bahir Dar Town. Urea (46% N) and Triple Super Phosphate (46% P_2_O_5_) was used as a source of N and phosphorus respectively.

**Table 2:** Lists and descriptions of the tested varieties

Name of Variety	Released year	Breeder/Maintainer	Altitude (AMSL)	Maturity days
Russet	2012	Greenline Trading PLC.	500–2800	90
Red coach	2015	Gawt International Business PLC.	500–2200	105
Jambar	2011	Jones Rick	500–2800	90
Bombay red	1980	MARC	700–2000	110–120

Where: EC, electrical conductivity; CEC, cation exchange capacity

### Experimental set-up and management

The treatments consisted of three hybrid onion varieties namely Red coach, Russet, Jambar and one open pollinated variety (Bombay red) as standard check and four N fertilizer rates (0, 41, 82 and 123 kg N ha^− 1^). The experiments were laid out using randomized complete block design (RCBD) in factorial arrangement with three replications following the procedures described by Gomez and Gomez [19]. The spacing between furrow, double rows and between plants was 40, 20 and 10 cm, respectively.

The amount of N, based on the treatment rates, was applied in the form of urea (46% N) and was first divided into two equal portions. The first half was applied 15 days after transplanting, and the remaining half was applied 45 days after transplanting in side-dressing method beside the plant rows. Triple superphosphate (46% P2O5) was used as a source of phosphorus and was applied at a rate of 92 kg ha^− 1^ P_2_O_5_ at the time of seedling transplanting uniformly for all experimental plots [20].

Transplanted seedlings were irrigated by using furrow irrigation at five days intervals until their establishment and at seven days intervals until maturity. To prevent the mixing of fertilizer rate, irrigation water was not passed from plot to plot. However, fifteen days before harvesting, irrigation was stopped for curing onion bulbs. The crop was harvested when 80% of the leaves turned yellow, dried and toppled of leaves, and attained full size of bulbs [21]. All seedling management and agronomic practices were performed as per the recommendations of EIAR [22].

### Data collection

#### Phenological parameters

Stand count percentage (%) was recorded by counting the plants that were successfully established in the central rows (considered more representative of the true yield potential of the plot) at harvest, and expressing the result as a percentage using the following equation [23].

$$\:\mathrm{S}\mathrm{t}\mathrm{a}\mathrm{n}\mathrm{d}\:\mathrm{C}\mathrm{o}\mathrm{u}\mathrm{n}\mathrm{t}\:\left(\mathrm{\%}\right)=\frac{\mathrm{N}\mathrm{u}\mathrm{m}\mathrm{b}\mathrm{e}\mathrm{r}\:\mathrm{o}\mathrm{f}\:\mathrm{p}\mathrm{l}\mathrm{a}\mathrm{n}\mathrm{t}\mathrm{s}\:\mathrm{e}\mathrm{s}\mathrm{t}\mathrm{a}\mathrm{b}\mathrm{l}\mathrm{i}\mathrm{s}\mathrm{h}\mathrm{e}\mathrm{d}\:}{\mathrm{N}\mathrm{u}\mathrm{m}\mathrm{b}\mathrm{e}\mathrm{r}\:\mathrm{o}\mathrm{f}\:\mathrm{p}\mathrm{l}\mathrm{a}\mathrm{n}\mathrm{t}\mathrm{s}\:\mathrm{i}\mathrm{n}\mathrm{i}\mathrm{t}\mathrm{i}\mathrm{a}\mathrm{l}\mathrm{l}\mathrm{y}\:\mathrm{p}\mathrm{l}\mathrm{a}\mathrm{n}\mathrm{t}\mathrm{e}\mathrm{d}}\mathrm x\;100$$

Days to maturity were recorded by counting the number of days elapsed from the date of seedling transplanted to a day at which more than 80% of the plants in a plot showed yellowing of leaves and the leaves dried and fall of for attained physiological maturity [6].

#### Growth parameters

Plants that developed flower stalks and exhibited premature bolting during vegetative growth were counted, and the bolting percentage was calculated as the ratio of bolted plants to the total number of plants in the plot [24]. Plant height was measured from the ground level to the tip of the longest leaf in ten randomly selected plants grown in the net plot area at physiological maturity using a tape meter [25]. The length of the longest leaf in ten randomly selected plants within the net plot area was measured in centimeters using a measuring tape at physiological maturity and recorded as leaf length [26]. The number of leaves per plant was determined by counting and averaging the total number of leaves from ten randomly selected plants in the net plot area at physiological maturity.

#### Yield parameters

The average bulb weight was measured by weighing ten randomly selected bulbs harvested from the net plot area using a sensitive balance. Bulb diameter was determined by measuring the midsection of ten randomly selected bulbs from the net plot area with a digital caliper [27]. Bulb length was recorded the mean length of ten randomly selected bulbs measured from the bottom to the top by using digital caliper [28]. Bulb neck diameter was recorded by measuring the average neck widths of ten randomly selected bulbs harvested from the net plot area using digital caliper [29]. Marketable bulb yield was recorded by weighing the total weight of healthy and free of mechanical and insect pest damages, and uniform in color bulbs harvested from net plot area using digital weight balance [29]. Unmarketable bulb yield was recorded by weighing the total weight of bulbs that were diseased, decayed, physically damaged and discolored bulbs harvested from the net plot area was measured in kilogram and expressed in percentage using the following equation.

$$\:\mathrm{U}\mathrm{n}\mathrm{m}\mathrm{a}\mathrm{r}\mathrm{k}\mathrm{e}\mathrm{t}\mathrm{a}\mathrm{b}\mathrm{l}\mathrm{e}\:\mathrm{b}\mathrm{u}\mathrm{l}\mathrm{b}\mathrm{s}\:\left(\mathrm{\%}\right)=\frac{\mathrm{W}\mathrm{e}\mathrm{i}\mathrm{g}\mathrm{h}\mathrm{t}\:\mathrm{o}\mathrm{f}\:\mathrm{u}\mathrm{n}\mathrm{m}\mathrm{a}\mathrm{r}\mathrm{k}\mathrm{e}\mathrm{t}\mathrm{a}\mathrm{b}\mathrm{l}\mathrm{e}\:\mathrm{b}\mathrm{u}\mathrm{l}\mathrm{b}\mathrm{s}}{\mathrm{T}\mathrm{o}\mathrm{t}\mathrm{a}\mathrm{l}\:\mathrm{w}\mathrm{e}\mathrm{i}\mathrm{g}\mathrm{h}\mathrm{t}\:\mathrm{o}\mathrm{f}\:\mathrm{b}\mathrm{u}\mathrm{l}\mathrm{b}\mathrm{s}}\:x\:100$$

The total bulb yield was obtained by the summation of marketable and unmarketable bulb yields [28].

#### Quality parameters

Harvest index was calculated as the ratio of total dry bulb yield to total dry biomass yield which was recorded from ten randomly selected plants after the bulbs harvested and cured, and expressed in percentage using the equation [30].

$$\:\mathrm{H}\mathrm{a}\mathrm{r}\mathrm{v}\mathrm{e}\mathrm{s}\mathrm{t}\:\mathrm{i}\mathrm{n}\mathrm{d}\mathrm{e}\mathrm{x}\:\left(\mathrm{H}\mathrm{I}\right)=\frac{\mathrm{w}\mathrm{e}\mathrm{i}\mathrm{g}\mathrm{h}\mathrm{t}\:\mathrm{o}\mathrm{f}\:\mathrm{d}\mathrm{r}\mathrm{y}\:\mathrm{b}\mathrm{u}\mathrm{l}\mathrm{b}\:\left(\mathrm{E}\mathrm{c}\mathrm{o}\mathrm{n}\mathrm{o}\mathrm{m}\mathrm{i}\mathrm{c}\:\mathrm{Y}\mathrm{i}\mathrm{e}\mathrm{l}\mathrm{d}\right)}{\mathrm{w}\mathrm{e}\mathrm{i}\mathrm{g}\mathrm{h}\mathrm{t}\:\mathrm{o}\mathrm{f}\:\mathrm{b}\mathrm{i}\mathrm{o}\mathrm{l}\mathrm{o}\mathrm{g}\mathrm{i}\mathrm{c}\mathrm{a}\mathrm{l}\:\mathrm{y}\mathrm{i}\mathrm{e}\mathrm{l}\mathrm{d}\:\left(\mathrm{a}\mathrm{b}\mathrm{o}\mathrm{v}\mathrm{e}\:\mathrm{a}\mathrm{n}\mathrm{d}\:\mathrm{b}\mathrm{e}\mathrm{l}\mathrm{o}\mathrm{w}\:\mathrm{g}\mathrm{r}\mathrm{o}\mathrm{u}\mathrm{n}\mathrm{d}\:\mathrm{d}\mathrm{r}\mathrm{y}\:\mathrm{w}\mathrm{e}\mathrm{i}\mathrm{g}\mathrm{h}\mathrm{t}\right)}x100$$

The total soluble solid (TSS) was determined after harvesting and curing from five randomly selected bulbs using digital refractometer following the procedures described by Tekeste et al. [27]. The dry matter content of bulbs (%) was calculated using the formula [31].

$$\:\mathrm{B}\mathrm{u}\mathrm{l}\mathrm{b}\:\mathrm{d}\mathrm{r}\mathrm{y}\:\mathrm{m}\mathrm{a}\mathrm{t}\mathrm{t}\mathrm{e}\mathrm{r}\:\mathrm{c}\mathrm{o}\mathrm{n}\mathrm{t}\mathrm{e}\mathrm{n}\mathrm{t}\mathrm{s}\:\left(\mathrm{\%}\right)=\frac{\mathrm{D}\mathrm{r}\mathrm{y}\:\mathrm{b}\mathrm{u}\mathrm{l}\mathrm{b}\:\mathrm{w}\mathrm{e}\mathrm{i}\mathrm{g}\mathrm{h}\mathrm{t}}{\mathrm{F}\mathrm{r}\mathrm{e}\mathrm{s}\mathrm{h}\:\mathrm{b}\mathrm{u}\mathrm{l}\mathrm{b}\:\mathrm{w}\mathrm{e}\mathrm{i}\mathrm{g}\mathrm{h}\mathrm{t}}x100$$

Before determining the dry matter content, five onion bulbs were randomly selected from each net plot, chopped into small pieces, and thoroughly mixed. From the composite sample, 50 g of chopped bulb tissue was taken from each plot and recorded as the fresh weight. The samples were then placed in an oven at 65 °C for 48 h until a constant weight was achieved. Subsequently, the dried samples were weighed using a digital balance and recorded as the dry weight.

#### Data analysis

Analysis of variance (ANOVA) was carried out using SAS version 9.2 software computer package programs [32], version 9.2. Mean separations were conducted using Least Significant Difference (LSD) at 1 and 5% probability level depending on the ANOVA results [19].

#### Economic analysis

The partial budget analysis was done for economic analysis of onion varieties with appropriate N fertilizer [33]. The cost of seeds, fertilizer and labor cost for their preparation were considered to calculate the total variable costs. Labor costs fluctuate with changes in the quantity of inputs used, depending on the intensity of management and the number of operations performed. For instance, the treatment that received 123 kg N ha⁻¹ required more labor than the control treatment. The gross benefit was calculated by multiplying the marketable yield downscaled by 10% with the field price of onion at the time of production. The net benefit was then calculated by subtracting the variable cost from the gross benefit. Dominance analysis was carried out by listing the treatments in an ascending order of total variable costs before calculating the marginal rate of return (MRR). According to CIMMYT [33], any treatment that has a net benefit less or equal to the previous treatment will be dominant and eliminated from further analysis. Following dominance analysis, the MRR was computed using the formula presented below.

$$\:\mathrm{M}\mathrm{R}\mathrm{R}\:\left(\mathrm{\%}\right)=\frac{\mathrm{C}\mathrm{h}\mathrm{a}\mathrm{n}\mathrm{g}\mathrm{e}\:\mathrm{i}\mathrm{n}\:\mathrm{N}\mathrm{e}\mathrm{t}\:\mathrm{B}\mathrm{e}\mathrm{n}\mathrm{e}\mathrm{f}\mathrm{i}\mathrm{t}\:}{\mathrm{C}\mathrm{h}\mathrm{a}\mathrm{n}\mathrm{g}\mathrm{e}\:\mathrm{i}\mathrm{n}\:\mathrm{T}\mathrm{o}\mathrm{t}\mathrm{a}\mathrm{l}\:\mathrm{V}\mathrm{a}\mathrm{r}\mathrm{i}\mathrm{a}\mathrm{b}\mathrm{l}\mathrm{e}\:\mathrm{C}\mathrm{o}\mathrm{s}\mathrm{t}}x100$$

## Results

### Phenological parameters

#### Stand count percentage

The main effects of variety and N fertilizer significantly (*P* < 0.001) influenced the stand count percentage of onion plants. However, the interaction between the two factors had no significant effect on stand count percentage. The highest stand count percentage (99.85%) was recorded for the Russet variety, while the lowest (94.88%) was observed for the Bombay Red variety (Table 3). As the rate of N fertilization increased from 0 to 41 kg ha⁻¹, the stand count percentage also increased. However, further increasing the N fertilizer rate beyond 41 kg ha⁻¹ did not significantly affect the stand count percentage.

**Table 3:** Main effects of varieties and N fertilizer on phonological and growth parameters of onion

Onion Varieties	Stand count (%)	Days to maturity (days)	Plant height (cm)	Leaf length (cm)	Leaf number
Bombay red	94.88^c^	113.17^a^	52.68^c^	43.72^c^	11.42^a^
Red coach	97.62^b^	106.17^b^	56.21^b^	48.68^b^	10.21^b^
Russet	99.85^a^	95.16^d^	58.89^a^	51.45^a^	10.28^b^
Jambar	97.63^b^	102.33^c^	60.53^a^	53.25^a^	10.35^b^
*P*-values	***	***	***	***	**
Nitrogen Rate					
0	95.19^b^	97.08^d^	50.33^d^	43.56^c^	9.40^c^
41	98.06^a^	102.08^c^	57.52^c^	49.10^b^	10.12^b^
82	98.66^a^	106.50^b^	59.34^b^	51.25^ab^	11.23^a^
123	98.06^a^	110.25^a^	61.12^a^	53.21^a^	11.50^a^
*P*-values	*	***	***	***	***
LSD (0.05)	2.15	3.78	1.78	2.35	0.69
CV (%)	2.65	4.36	3.75	5.74	7.86
SE±	0.31	0.67	0.27	0.36	0.09

Means followed by the same letter(s) within a column are not significantly different. Letters a,b, c, and d indicate the results of an LSD test comparing group means to identify statisticallydifferent groups. The letters are assigned in descending order of the means

*** Very highly significant at P < 0.001; * = significant at P < 0.05; LSD, least significancedifference; CV, Coefficient of variation and SE, Standard error

#### Days to maturity

The ANOVA indicated that the longest days to bulb maturity (113.17 days) were observed from Bombay red variety, whereas the shortest days to bulb maturity (95.16 days) were observed in Russet variety (Table 3). Regarding N fertilizer, onion plants grown with 123 kg N ha^− 1^ required the greatest number of days (110.25 days) to reach maturity, while the control treatments required fewest days (97.08 days) to maturity.

### Growth parameters

#### Bolting percentage

The ANOVA indicated that both the main effects and the interaction effects of varieties and N fertilizer significantly influenced the bolting percentage of onion. Accordingly, the highest bolting percentage (25.68%) was recorded for the treatment combination of Bombay Red with the unfertilized treatment. On the other hand, the hybrid onion varieties, regardless of the N fertilizer rate, did not exhibit bolting (Table 4). This might be associated with genetic variation among the varieties.

**Table 4:** Interaction effects of varieties and N fertilizer on bolting percentage of onion

Onion Varieties	0 kg *N* ha^− 1^	41 kg *N* ha^− 1^	82 kg *N* ha^− 1^	123 kg *N* ha^− 1^
Bombay red	25.68^a^	19.08^b^	12.50^c^	7.45^d^
Red coach	0.00	0.00	0.00	0.00
Russet	0.00	0.00	0.00	0.00
Jambar	0.00	0.00	0.00	0.00
*P*-values				***
LSD (0.05)				0.71
CV (%)				10.34
SE±				0.05

Means followed by the same letter(s) within a column are not significantly different. Letters a,b, c, and d indicate the results of an LSD test comparing group means to identify statisticallydifferent groups. The letters are assigned in descending order of the means

*** Very highly significant atP< 0.001; LSD, least significance difference; CV, Coefficient ofvariation and SE, Standard error

#### Plant height

Plant height in onion varieties was significantly (*P* < 0.0001) influenced by the main effects of variety and N fertilizer. However, the interaction effects between these factors did not significantly influence this parameter. The results showed that the tallest plants (60.53 cm) were obtained from Jambar variety, while the shortest plants (52.68 cm) were observed in the Bombay Red variety, as indicated in Table 3. Regarding fertilizer, the longest plant height (61.12 cm) was recorded at the highest N fertilizer rate, while the shortest plant height (50.33 cm) was observed in the control treatment.

#### Leaf length

The ANOVA results showed that the main effects of variety and N fertilizer significantly influenced the leaf length of onion plants, whereas their interaction effect was not of both factors were not significant. The longest leaf length (53.25 cm) was recorded for the Jambar variety, while the shortest (43.72 cm) was recorded for the Bombay Red variety (Table 3). Similarly, the longest leaf length (53.21 cm) was obtained with the highest N fertilizer rate, whereas the shortest (43.56 cm) was observed in the control treatment (no fertilizer).

#### Leaf number

The ANOVA results indicated that the main effects of variety and N fertilizer were significantly (*P* < 0.001) influential on the leaf number of onion plants. However, the interaction effect between the two factors did not significantly influence this parameter. The highest leaf number (11.42) was recorded for the Bombay Red variety, whereas the lowest (10.21) was recorded in the Red Coach variety (Table 3). Regarding N fertilization, the maximum leaf number per plant (11.50) was obtained at the highest N fertilizer application, while the minimum (9.40) was observed in the control treatment (no N fertilizer) (Table 3).

### Yield parameters

#### Bulb weight

The ANOVA indicated that the highest bulb weight (165.87 g) was obtained from the treatment combination of the Russet variety with 82 kg N ha^− 1^, while the lowest bulb weight (72.33 g) was recorded from the treatment combination of the Bombay Red variety with no N fertilizer (Table 5).

**Table 5:** Yield components of onion as influenced by the interaction effect of variety and N fertilizer

Onion varieties	Nitrogen rate (kg ha^− 1^)	Bulb weight (g)	Bulb diameter (cm)
Bombay red	0	72.33^k^	4.85^j^
	41	82.43^j^	5.27^i^
	82	94.57^i^	6.28^h^
	123	96.83^i^	6.36^h^
Red coach	0	114.21^h^	6.15^h^
	41	143.37^e^	6.76^efg^
	82	150.82^cd^	7.28^bc^
	123	147.47^de^	6.87^edf^
Russet	0	134.30^f^	6.49^fgh^
	41	155.80^bc^	7.31^bc^
	82	165.87^a^	7.89^a^
	123	158.50^b^	7.50^b^
Jambar	0	127.08^g^	6.43^gh^
	41	152.60b^cd^	7.20^cbd^
	82	156.74^bc^	7.47^b^
	123	149.81^cde^	7.05^cde^
*P*-values		**	**
CV (%)		4.30	3.40
SE±		0.67	0.03

Means followed by the same letter(s) within a column are not significantly different. Letters a,b, c, d, e, f, g, h, i, j and k indicate the results of an LSD test comparing group means to identifystatistically different groups. The letters are assigned in descending order of the means andascending alphabetical order

** highly significant at P < 0.01; LSD, Least Significance Difference; CV, Coefficient ofvariation and SE, Standard error

#### Bulb diameter

The ANOVA revealed that the bulb diameter of onion was significantly (*P* < 0.001) affected by both the main effects and the interaction effect of variety and N fertilizer. The interaction results indicated that the maximum bulb diameter (7.89 cm) was recorded for the treatment combination of the Russet variety with 82 kg N ha^− 1^, while the minimum bulb diameter (4.85 cm) was obtained from the treatment combination of the Bombay Red variety with unfertilized treatment, as shown in Table 5.

#### Bulb neck diameter

The ANOVA results revealed that the bulb neck diameter was significantly (*P* < 0.0001) influenced by the main effects of variety and N fertilizer. However, the interaction effect of the two factors did not influence bulb neck diameter in onion. Accordingly, the highest bulb neck diameter (1.52 cm) was recorded for the Bombay Red variety, whereas the lowest (1.29 cm) was obtained from the Russet variety (Table 6). In the present study, the highest bulb neck diameter (1.56 cm) was obtained from the treatment with the highest N fertilizer rate, whereas the lowest bulb neck diameter (1.26 cm) was recorded from the treatment that received no N fertilizer.

**Table 6:** Main effects of variety and N fertilizer on yield parameters of onion

Onion Varieties	Bulb neck diameter (cm)	Bulb length (cm)	Unmarketable bulb yield (%)
Bombay red	1.52^a^	5.12^b^	0.86^a^
Red coach	1.50^a^	6.58^a^	0.61^b^
Russet	1.29^c^	6.67^a^	0.37^d^
Jambar	1.40^b^	6.63^a^	0.48^c^
*P*-values	***	***	***
Nitrogen Rate			
0	1.26^c^	5.72^c^	0.89^c^
41	1.39^b^	6.08^b^	0.62^b^
82	1.50^a^	6.64^a^	0.44^a^
123	1.56^a^	6.56^a^	0.37^a^
*P*-values	***	***	***
LSD (0.05)	0.09	0.35	0.07
CV (%)	7.89	6.78	10.62
SE±	0.02	0.05	0.02

Mean followed by the same letter(s) within a column are not significantly different. Letters a, b,c and d indicate the results of an LSD test comparing group means to identify statisticallydifferent groups. The letters are assigned in descending order of the means

*** Very highly significant at P < 0.001; LSD, Least significance difference; CV, Coefficient ofvariance; and SE, Standard error

#### Bulb length

Bulb length of onion was significantly (*P* < 0.0001) influenced by the main effects of variety and N fertilizer. However, the interaction between the two factors did not significantly influence this parameter. The highest bulb length (6.67 cm) was recorded for the Russet variety, whereas the lowest bulb length (5.12 cm) was obtained from the Bombay Red variety (Table 6). With respect to N fertilizer, the highest bulb length (6.64 cm) was obtained from the application of 82 kg ha^− 1^ of N fertilizer, while the lowest bulb length (5.72 cm) was recorded in the treatment without N fertilizer (Table 6).

#### Marketable bulb yield

**Fig. 2:**
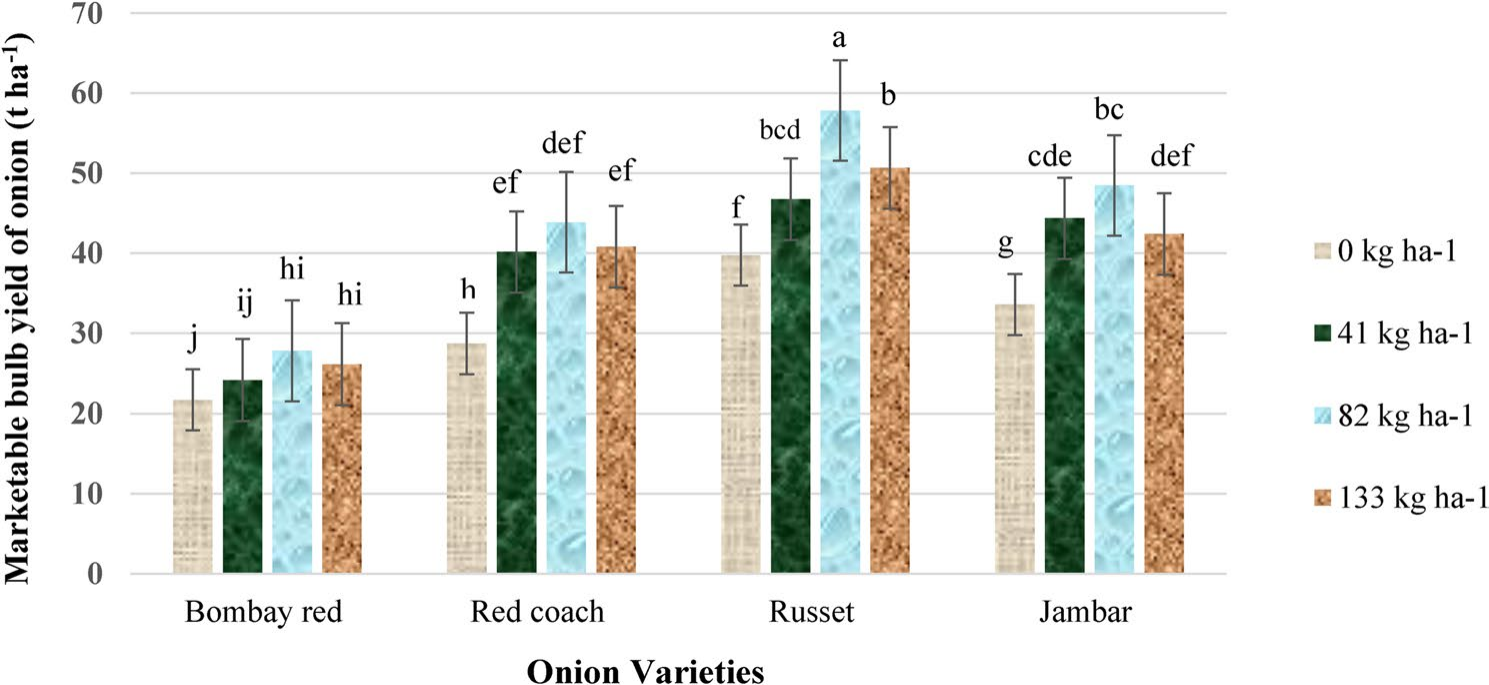
Interaction effect of variety and N fertilizer on marketable bulb yield of onion. Note: Mean values followed by the same letter(s) are not significantly different at *P* < 0.01. Letters a, b, c, d, e, f, g, h, i and j indicate the results of an LSD test comparing group means to identify statistically different groups. The letters are assigned in descending order of the means and ascending alphabetical order. t ha⁻¹, tonne per hectare; kg ha⁻¹, kilogram per hectare

The ANOVA showed that both the main effects (*P* < 0.0001) and interaction effects (*P* < 0.001) of variety and N fertilizer significantly influenced the marketable bulb yield of onion. The highest marketable bulb yield (57.84 t ha^− 1^) was obtained from the combination of the Russet variety with 82 kg N ha^− 1^, whereas the lowest marketable bulb yield (21.74 t ha^− 1^) was obtained from the combination of the Bombay Red variety with no N fertilizer (Fig. 2).

#### Unmarketable bulb yield

The ANOVA revealed that the highest proportion unmarketable yield (0.86%) was recorded for the Bombay Red variety, whereas the lowest (0.37%) was observed for the Russet variety (Table 6). Regarding N fertilizer rates, the lowest unmarketable bulb yield was observed at 123 kg N ha⁻¹. The relatively higher unmarketable yield in the unfertilized treatments might be attributed to suboptimal N supply, which likely resulted in fewer bulbs and poor bulb development.

#### Total bulb yield

**Fig. 3:**
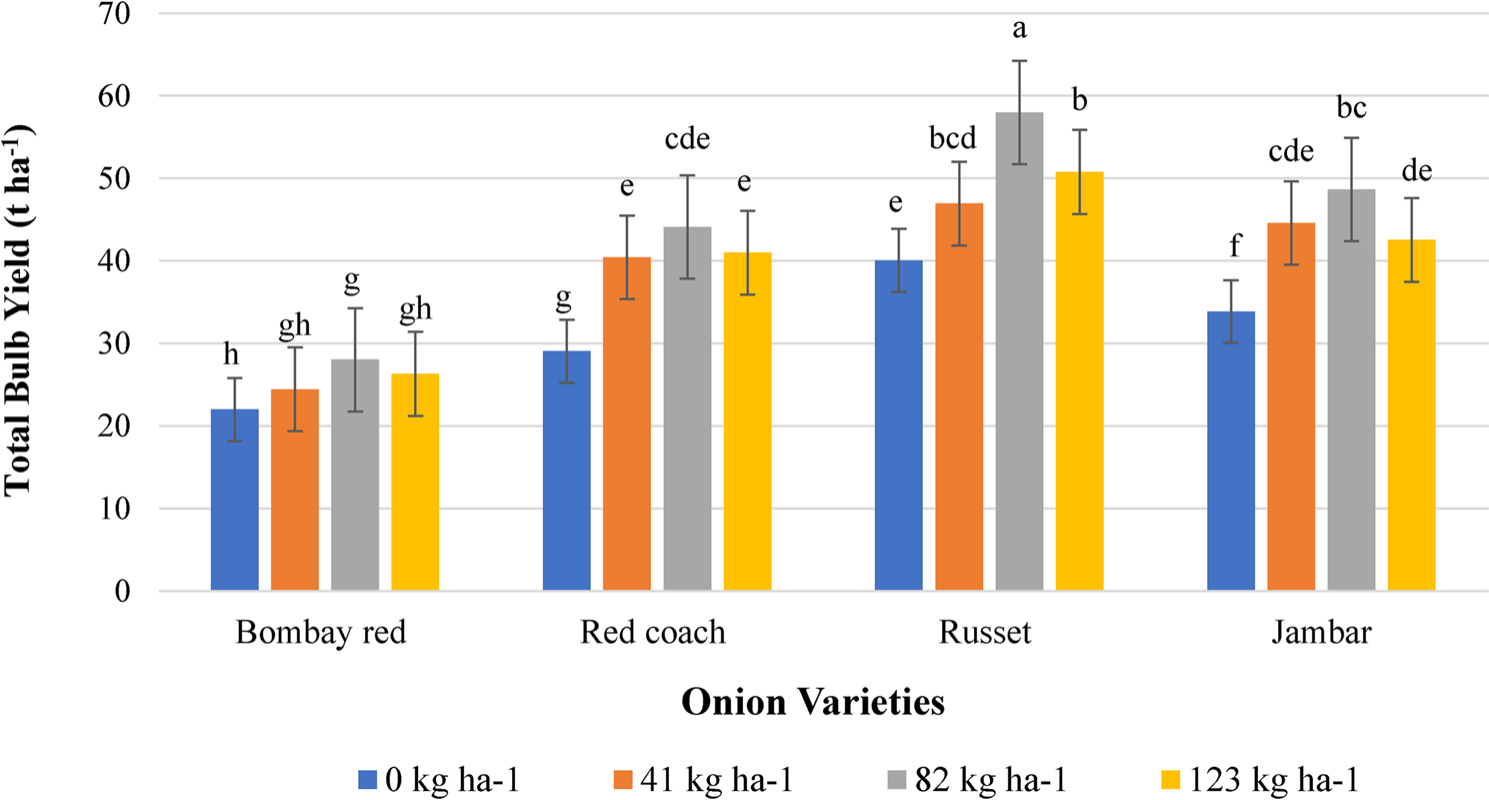
Interaction effect of variety and N fertilizer on total bulb yield of onion. Note: Mean values followed by the same letter(s) are statistically similar at P < 0.01. Letters a, b, c, d, e, f, g and h indicate the results of an LSD test comparing group means to identify statistically different groups. The letters are assigned in descending order of the means and ascending alphabetical order. t ha⁻¹, tone per hectare; kg ha⁻¹, kilogram per hectare

The ANOVA results indicated that the total bulb yield of onion was highly significantly affected (*P* < 0.0001) by both the main effects and the interaction between variety and N fertilizer. The maximum total bulb yield (57.98 t ha⁻¹) was obtained from the Russet variety combined with 82 kg N ha⁻¹, whereas the minimum yield (21.99 t ha⁻¹) was recorded for the Bombay Red variety grown without N fertilizer. Overall, total bulb yield for all onion varieties increased with increasing N fertilizer rate (Fig. 3).

### Quality parameters

#### Harvest index

The ANOVA revealed that the highest harvest index of onion was obtained from the Russet variety, whereas the lowest was recorded for the Bombay Red variety, as shown in Table 7. With respect to N fertilization, the greatest harvest index was observed at a N application rate of 82 kg ha⁻¹, while the lowest value occurred in the treatment without N application. The increase in harvest index with N fertilization might be attributed to enhanced bulb dry weight and higher marketable yield.

**Table 7:** Main effects of varieties and N fertilizer rates on the quality parameters of onion

Onion Varieties	Harvest Index (%)	Total soluble solid (^°^Brix)	Dry matter Content (%)
Bombay red	89.32^d^	11.25^a^	12.28^a^
Red coach	92.23^c^	8.67^c^	9.56^c^
Russet	98.16^a^	8.58^c^	9.50^c^
Jambar	95.18^b^	9.42^b^	10.26^b^
*P*-values	***	***	***
Nitrogen Rate			
0	90.85^c^	8.75^c^	9.60^c^
41	93.66^b^	9.25^b^	10.27^b^
82	96.21^a^	9.83^a^	10.85^a^
123	94.16^b^	10.08^a^	10.88^a^
*P*-values	***	***	***
LSD (0.05)	1.78	0.48	0.56
CV (%)	2.29	6.18	6.48
SE±	0.25	0.07	0.08

Mean followed by the same letter(s) within a column are not significantly different. Letters a, b,c and d indicate the results of an LSD test comparing group means to identify statisticallydifferent groups. The letters are assigned in descending order of the means

*** Very highly significant at P < 0.001; LSD, Least Significance Difference; CV, Coefficient ofVariance; and SE, Standard Error

#### Total soluble solid

The ANOVA showed that the highest TSS content in onion was recorded for the Bombay Red variety (Table 7). Regarding N fertilizer rates, the highest TSS value was observed at the higher N application levels, whereas the lowest TSS was recorded in the treatment without N fertilizer.

#### Dry matter contents

The ANOVA indicated that the Bombay Red variety exhibited the highest bulb dry matter content (Table 7). With respect to N fertilization, the highest bulb dry matter content was observed in treatments receiving higher N rates, whereas the lowest value was recorded in control (unfertilized) treatments.

#### Partial budget analysis

A partial budget analysis was conducted following the methodology described by CIMMYT [33], considering the costs of fertilizer, seed, and labor as variable costs. Gross income was calculated from the marketable bulb yield, which was adjusted downward by 10% to better reflect farmers’ yields. The sale price of onion during the experimental period was 0.25 USD kg^− 1^, and seed costs varied among varieties. According to the results of the partial budget analysis, the highest net benefit (12,419.87 USD ha⁻¹) was obtained from the combination of the Russet variety with 82 kg N ha⁻¹. This result is likely associated with the higher marketable bulb yield produced by this treatment compared with the other treatment combinations (Table 8).

**Table 8:** Economic analysis of onion as influenced by varieties and N fertilizer

Varieties	*N* fertilizer (kg)	SC (USD)	LC (USD)	FC (USD)	TVC (USD)	MY (t ha^− 1^)	AMY (t ha^− 1^)	GB (USD)	Net benefit (USD)
Russet	0	533.33	0	0	533.33	39.77	35.79	8947.5	8414.17
Russet	41	533.33	6.25	23.40	562.98	46.75	42.07	10517.5	9954.52
Russet	82	533.33	12.5	46.79	592.63	57.84	52.05	13012.5	12419.87
Russet	123	533.33	18.75	70.19	622.27	50.66	45.59	11397.5	10775.23
Jambar	0	466.67	0	0	466.67	33.60	30.24	7560	7093.33
Jambar	41	466.67	6.25	23.40	496.31	44.37	39.93	9982.5	9486.19
Jambar	82	466.67	12.5	46.79	525.96	48.47	43.62	10,905	10379.04
Jambar	123	466.67	18.75	70.19	555.61	42.40	38.16	9540	8984.39
Red coach	0	433.33	0	0	433.33	28.75	25.87	6467.5	6034.17
Red coach	41	433.33	6.25	23.40	462.98	40.17	36.15	9037.5	8574.52
Red coach	82	433.33	12.5	46.79	492.63	43.87	39.48	9870	9377.37
Red coach	123	433.33	18.75	70.19	522.27	40.80	36.72	9180	8657.73
Bombay red	0	83.33	0	0	83.33	21.74	19.57	4892.5	4809.17
Bombay red	41	83.33	6.25	23.40	112.98	24.19	21.77	5442.5	5329.52
Bombay red	82	83.33	12.5	46.79	142.63	27.82	25.04	6260	6117.37
Bombay red	123	83.33	18.75	70.19	172.27	26.15	23.53	5882.5	5710.23

SC , Seed cost; LC, Labor cost; FC, Fertilizer cost; TVC, Total variable cost; MY, Marketablebulb yield; AMY, Adjusted marketable yield; GB, Gross benefit; seed costs (Bombay red, Redcoach, Jambar and Russet varieties were 20.83, 108.33, 116.66 & 133.33 USD kg respectively); Fertilizer cost = 0.15 USD kg ; farm get price = 0.25 USD kg− 1

#### MRR

The MRR was analyzed to compare the cost and net benefit of the treatments using the procedure described by CIMMYT [33]. The treatments were arranged in ascending order of total variable costs, and any treatment with a net benefit less than or equal to that of the preceding treatment was considered dominated and excluded from further analysis. Accordingly, the highest MRR of 3,061.09% was achieved by the combination of the Russet variety with 82 kg N ha⁻¹ (Table 9).

**Table 9:** MRR of onion as influenced by the varieties and N fertilizers

Treatment combinations	TVC (USD)	Net benefit (USD)	Dominance analysis	MRR (%)	Rank
Bombay red × 0	83.33	4809.17			
Bombay red × 41	112.98	5329.52		1754.97	6
Bombay red × 82	142.63	6117.37		2657.17	5
Bombay red × 123	172.27	5710.23	D		
Red coach ×0	433.33	6034.17	D		
Red coach ×41	462.98	8574.52		767.02	7
Red coach ×82	492.63	9377.37		2707.76	4
Red coach × 123	522.27	8657.73	D		
Jambar× 0	466.67	7093.33	D		
Jambar× 41	496.31	9486.19		2957.07	3
Jambar× 82	525.96	10379.04		3011.30	2
Jambar× 123	555.61	8984.39	D		
Russet × 0	533.33	8414.17	D		
Russet × 41	562.98	9954.52	D		
Russet × 82	592.63	12419.87		3061.09	1
Russet × 123	622.27	10775.23	D		

StartFragment D, Dominance; MRR, marginal rate of return; TVC, Total variable costEndFragment

## Discussion

### Effects of N fertilizer rate on the phenological development of onion varieties

The observed difference in stand count percentage among the evaluated onion varieties can be primarily attributed to genetic variations. This finding is consistent with the report of Fikre and Awoke [11], who similarly demonstrated that stand count percentage at harvest was influenced by varietal differences in onions due to their genetic makeup. Furthermore, the markedly higher stand count percentages recorded at N application rates of 41 kg ha⁻¹ and higher could be attributed to reduced competition among onion plants for growth factors due to the availability of N. These results are supported by the findings of Guesh [28], who noted the lowest bulb yields and, by extension, poorest stand establishment in unfertilized control plots, underscoring the critical role of N nutrition in supporting plant survival and population density through the cropping cycle.

The variation in days to physiological bulb maturity among the tested onion varieties can largely be attributed to variation in genotypes and the reaction of varieties with the environment. This observation is in close agreement with the findings of Fikre and Awoke [11] and Gebremedhn et al. [34], who observed that the differences in the length of the bulbing phase among onion cultivars are primarily governed by inherent genetic makeup. Increasing the application of N fertilizer prolonged the days to bulb maturity of onion. Onion plants grown at the higher rates of N required a greater number of days for maturity than the lower rates of N. This prolongation of the maturity period at elevated N supply is most likely associated with extended vegetative growth and sustained canopy duration, which allows continued photosynthetic activity and delays the onset of bulb ripening. The present results align well with those reported by Guesh [28], who similarly found that the highest N dose markedly prolonged the period to bulb maturity relative to lower N rates and the control treatment, further confirming the strong influence of N availability on the transition from vegetative to reproductive development in onion.

### Influence of N fertilizer rate on vegetative growth parameters of onion varieties

In the present study, none of the tested hybrid onion varieties showed any bolting, irrespective of N fertilizer rate (Table 4). This complete lack of premature flowering is likely a reflection of strong genetic resistance to bolting inherent in the selected F1 hybrid genotypes. Our observation agrees sharply with earlier studies, where the traditional open-pollinated cultivar Bombay Red exhibited the highest bolting percentage precisely when no N was applied [24, 35]. In onion, the decision to bolt is driven primarily by environmental signals, especially prolonged exposure to low temperatures (vernalization) during the juvenile stage together with a suitable photoperiod, while mineral nutrition has only a secondary influence [36]. In the absence of sufficient chilling (typically 4–10 °C for an extended period) or when day-length is inappropriate, floral initiation simply does not occur [37]. Given that breeding for minimal bolting is a key objective in hybrid onion programs for warm regions [38], the zero-bolting recorded here is most likely reflects the combined effects of inadequate vernalizing temperatures during the sensitive early growth phase, good adaptation of the hybrids to the local thermal and photoperiod regime, and their inherently low genetic tendency to bolt – rather than any direct effect of the N treatments.

The significant differences in plant height were observed among the tested onion varieties, which can be primarily ascribed to their genetic potential and response to environmental conditions. These findings corroborate earlier work by Fikre and Awoke [11] and Gebremedhn et al. [34], who reported that the observed differences in onion plant height are attributed to genotypic differences among varieties. Application of increasing rates of N fertilizer resulted in a consistent and marked increase in plant height across varieties. This positive response is attributable to the critical role of N in promoting cell division, cell elongation, and overall vegetative growth, ultimately leading to an expanded leaf area and greater photosynthetic capacity. The tallest plants were consistently recorded at the highest N level, a result that aligns closely with the observations of Weldemariam et al. [39], who reported that the longest plant height was obtained with the highest N fertilizer application. These results underscore the importance of adequate N nutrition in maximizing vegetative growth and canopy development in onion.

The difference in leaf length among onion varieties might be due to their inherent genetic variations, which affected the leaf length. This finding aligns closely with the work of Walle et al. [24], who found that the genetic makeup of varieties accounted for the differences in the leaf length of onions. On the other hand, the noticeable increase in leaf length with rising N fertilizer rates can be explained by nitrogen’s critical role in promoting cell division, cell elongation, and overall vegetative growth, ultimately expanding the plant’s photosynthetic surface area. These results are consistent with those reported by Muluneh et al. [6] and Abbas et al. [40], who also observed that higher doses of N fertilizer led to significantly longer leaves in onion crops. In onion plants, longer leaves generally translate into a bigger overall leaf surface area the part of the plant that catches sunlight and drives growth. When N is plentiful, the plants can produce more chlorophyll (giving leaves that rich green color) and keep their leaves healthy and functional for a longer time instead of letting them yellow and die off early [41].

The significant variation in the leaf number across the onion varieties most likely stem from their unique genetic backgrounds, some varieties are simply wired to put out more leaves than others. The results of the present study are consistent with the findings of Boukary et al. [26], who also concluded that the variation in the number of leaves per plant between onion varieties was attributed to their genetic makeup. On the other hand, the maximum number of leaves per plant obtained with higher N fertilizer application might be due to nitrogen’s role in improving the production of new shoots and the steady emergence of new leaves during the vegetative growth of plants, which in turn directly increases the leaf number of onions. The more N the plants received, the more leaves they kept producing. These results are in agreement with the findings of other researchers, who reported the highest number of leaves per plant was consistently achieved at the highest N application levels [25].

### Effects of N fertilizer rates on yield components of onion varieties

The clear differences in bulb weight between onion varieties most likely come down to genetic variation, differing responses to N, and their adaptability to prevailing environmental conditions. The improvement in bulb weight for the Russet variety in response to optimum N fertilizer can be attributed to its ability to mobilize and utilize N effectively [22]. This increased the production and allocation of assimilates to the bulbs, resulting in higher bulb weight. The present study is in agreement with the findings of Khan et al. [42], who indicated that onion varieties differed in bulb weight due to differences in genetic makeup and their response to N fertilization.

The noticeable difference in bulb diameters among the onion varieties can largely be attributed to the direct effects of genetic variation and to the differing responses each variety to N fertilizer application. The present investigation is in agreement with the findings of Kumar et al. [31] and Benti [35], who reported that differences in bulb diameter are driven by the genetic potential of the varieties and by their varying responses to N fertilization.

The significant difference in bulb neck diameters among onion varieties most likely come down to the effect of genetic variation, which influenced the bulb neck diameters. This matches exactly what Shoba et al. [43], found when they pointed the finger at genotypic differences as the main reason for variation in neck thickness. On the other hand, the development of a thicker bulb neck diameter with higher N fertilizer is attributed to the fact that when some proportion of the bulb fails to complete bulb formation, the leaves continue their growth due to N fertilization. This is precisely what Mandefro et al. [44] observed: the highest N treatments consistently produced the widest neck diameters. In short, while genetics set the baseline neck thickness, too much N can push the neck wider by delaying maturity and keeping the foliage active when it should be winding down.

The variation in bulb length among the tested onion varieties was primarily driven by their genetic constitution—some varieties are simply predisposed to develop longer, more elongated bulbs than others. This observation is fully consistent with the results of Birtukan et al. [29], who reported that the bulb length of onion was significantly influenced by the genotype of the varieties. On the other hand, the longer bulbs achieved with optimal N fertilization highlight the nutrient’s powerful influence on bulb development. N enhances metabolic processes, increases assimilate and dry matter production, and consequently leads to increased bulb length. These findings align closely with those of Tekeste et al. [27], who also recorded substantial increases in bulb length when onions were supplied with the right amount of N. Thus, while genetics establish the basic framework for bulb shape and size, a well-balanced N supply provides the metabolic boost needed for the bulb to fully express its length potential.

The marketable bulb yield of onion increased with higher N fertilizer application. The observed difference in marketable bulb yield of onion can be attributed to genetic variation and agro-ecological adaptations. The highest marketable bulb yield of the Russet variety with optimum N fertilizer is also associated with an increase in harvest index, bulb weight, and bulb diameter, implying that these are major determinants of bulb yield in onion. The present findings are in concordance with the findings of Rageb et al. [30], who stated that the varying yield response of onion varieties was observed with different N fertilization levels.

The proportion of unmarketable bulbs varied noticeably among the onion varieties, and this can largely be explained by their inherent genetic differences. Some varieties are simply more prone to producing bulbs with defects—such as splitting, doubling, thick necks while others naturally yield a cleaner, more uniform crop. These built-in genetic tendencies directly influence the percentage of bulbs that fail to meet market standards, even under the same growing conditions. The results of this study align with the findings of Fikre and Awoke [11], who confirmed that unmarketable bulb yield in onion is influenced by varietal differences and adaptability to the growing environment. On the other hand, the highest unmarketable bulb yield in unfertilized treatments of onion might be due to a suboptimal N supply, which led to lower bulb numbers and poor bulb formation. These results are consistent with the findings of Gebretsadik and Dechassa [45], who reported that higher N fertilizer application reduced the unmarketable bulb yield of onion compared to control treatments.

The differences between onion varieties could be attributed to their genetic variations, which influence plant growth and indirectly determine bulb size by affecting carbohydrate synthesis and storage in bulbs. The highest total bulb yield observed in the Russet variety with the optimum N fertilizer level was also associated with an increase in average bulb weight, indicating that bulb weight is a major determinant of total yield in onions. This finding suggests that the Russet variety has a higher nutrient utilization efficiency, better adaptability, and superior yield performance compared to the other onion varieties. The results of this study align with the findings of Walle et al. [24], who confirmed that onion varieties exhibit different total bulb yield potentials due to their response to N fertilization, genetic variation, and the interaction between genotype and environmental factors.

### Quality attributes of onion as influenced by N fertilizer and variety

Harvest index, the proportion of total dry matter that ends up as marketable bulbs varied considerably among the onion varieties, and once again playing the dominant role. The observed difference in harvest index among onion varieties can be attributed to their genetic makeup. A higher harvest index was associated with the production of larger bulb weights, whereas a lower harvest index resulted from smaller bulb weights relative to the above-ground biomass. The results of this study are consistent with the findings of Walle et al. [24], who reported that variations in the harvest index were due to the genetic differences among onion varieties. Additionally, the improvement in harvest index could be linked to an increase in the photosynthetic area, which enhances the production and partitioning of assimilates to the bulbs. These findings align with the study by Tsegaye et al. [23], who reported that the highest harvest index was obtained with higher N fertilizer application compared to the control.

N fertilizer has a significant effect on the TSS of onion varieties. This response may be partly related to genetic differences among the varieties. The present findings are consistent with those of Muluneh et al. [6] and Tiru et al. [46] who reported that the increase in TSS with higher N application may be associated with the role of N in enhancing chlorophyll content and plant dry weight, which in turn improves the TSS content of onion bulbs. These results are in agreement with the findings of Muluneh et al. [6]. Similarly, Al-Fraihat [47] reported that increasing the N fertilizer rate from 100 to 200 kg N ha^− 1^ increased the TSS from 13.75% to 14.70%. Morsy et al. [48] also reported that the application of 120 kg ha^− 1^ N led to the highest values of TSS, whereas the application of 90 kg N ha^− 1^ resulted in the lowest value of TSS. Generally, high-yield varieties with larger bulb sizes (higher bulb weights) have lower TSS contents than varieties with small bulbs and lower yields [21].

Increasing the N rate has been reported to enhance plant growth and development by stimulating photosynthetic activity and dry matter production [49], and a similar mechanism may explain the trends observed in the present study. The observed variation in bulb dry matter content among onion varieties is likely associated with inherent genetic differences that govern their efficiency in assimilate production and partitioning. The increase in dry matter content with higher N fertilization may be attributed to the role of N in promoting assimilate synthesis and accumulation, thereby increasing bulb dry weight. These findings are in line with the results of Muluneh et al. [6], who also reported a positive relationship between N application and dry matter content in onion.

## Conclusion and recommendations

This study was undertaken to evaluate the performance of promising hybrid onion varieties under varying N fertilizer rates and to identify the most productive and economically viable combination for smallholder farmers in Northwestern Ethiopia. The results fully achieved these objectives by demonstrating that both variety selection and N management exert highly significant influences on phenological, growth, yield components, and bulb quality. Among the tested combinations, the hybrid variety Russet, supplied with 82 kg N ha⁻¹, consistently recorded the highest marketable bulb yield (57.84 t ha⁻¹), the largest bulb size, and superior quality attributes. Partial budget analysis confirmed the economic superiority of this treatment, delivering the highest net benefit (12,419.87 USD ha⁻¹) and an exceptionally high MRR (3,061.09%), making it a highly attractive option for resource-limited farmers. Therefore, for the agro-ecological conditions of Northwestern Ethiopia, the integration of the Russet hybrid with an application of 82 kg N ha⁻¹ is recommended as the optimum package for maximizing both productivity and profitability of onion production. This finding is particularly relevant for the Amhara region and other mid- to high-altitude areas of Ethiopia and East Africa with similar short-day tropical environments, where hybrid onions are rapidly replacing traditional varieties. Adoption of this variety–N combination has the potential to significantly boost household income, improve food security, and enhance the competitiveness of smallholder onion producers in domestic and regional markets. Nevertheless, because genotype × environment interactions can be substantial in onion, multi-location and multi-season trials across representative onion-growing districts are strongly recommended to confirm the stability of Russet’s superior performance and the robustness of the 82 kg N ha⁻¹ recommendation before large-scale extension efforts are implemented. Such validation will further increase farmer confidence and accelerate the uptake of this high-yielding, economically rewarding production package.

## Data Availability

The datasets used and/or analyzed during the current study are available from the corresponding author on reasonable request.

## References

[1] Gessesew WS, Woldetsadik K, Wassu M. Growth parameters of onion (*Allium Cepa* L. var. Cepa) as affected by nitrogen fertilizer rates and Intra-row spacing under irrigation in Gode, South-Eastern Ethiopia. Agric Forestry Fisheries. 2015;4(6):239. 10.11648/j.aff.20150406.11.

[2] Griffiths G, Trueman B, Crowther B, Thomas B, Smith T. Onions - A global benefit to health. John Wiley and Sons, Ltd. 2002. 10.1002/ptr.1222 12410539

[3] Griffiths G, Trueman L, Crowther T, Thomas B, Smith B. Onions—A global benefit to health. Phytother Res. 2002;16(7):603–15. 10.1002/ptr.1222. 12410539 10.1002/ptr.1222

[4] Raemaekers HR. Crop Production in Tropical Africa. DGIC Ministry of Foreign affairs, External Trade and International cooperation, Brussels, Belgium. 2001;455(ISBN:90-806822-1-7).

[5] Raemaekers RH, editor. Crop Production in Tropical Africa. DGIC, Ministry of Foreign Affairs, External Trade and International Cooperation, Brussels, Belgium. 2001.

[6] NHRDF. Onion crop details. 2009. http://nhrdf.org/pMedicinalValues.o.php.

[7] Lemma D, Shimeles A, Selamawit K, Chimdo A. The vegetable seed sectors in ethiopia: current status and future prospects. EHSS Proc Inaugural Third Natl Hortic Workshop Ethiopia. 2006;1:103–9.

[8] Muluneh B, Alli M, Amsalu N. Effects of different level of nitrogen fertilizer application on Growth, Yield, quality and storage life of onion (Allium *Cepa* L.) at Jimma, South West Ethiopia. J Nat Sci Res. 2019;9(10):2224–3186. 10.7176/JNSR/9-10-05.

[9] Melkamu A, Fentahun T, Solomon B, Belayneh A. Amhara Region Horticultural Development Strategy. 2015;(2015–2019). https://www.researchgate.net/publication/318876775.

[10] Yeshiwas Y, Alemayehu M, Adgo E. The rise and fall of onion production; its multiple constraints on pre-harvest and post-harvest management issues along the supply chain in Northwest Ethiopia. Heliyon. 2023;9(5). 10.1016/j.heliyon.2023.e15905. 10.1016/j.heliyon.2023.e15905 PMC10192415 37215801

[11] Kitila C, Abdisa A, Soressa S. Growth and bulb yield of some onion (Allium Cepa L.) varieties as influenced by NPS fertilizer at Dambi Dollo university research site, Western Ethiopia. Cogent Food Agric. 2022;8:2097606. 10.1080/23311932.2022.2097606.

[12] Brewster JL. Onions and other vegetable Alliums. CABI international vol. UK: II, Biddles Ltd, King’s Lynn; 2008. p. 455.

[13] Fikre G, Awoke M. Adaptation and evaluation of improved onion (Allium *cepa*) varieties at Arba Minch, Southern Ethiopia. Asian J Plant Sci Res. 2021;11(8):264–8. https://www.researchgate.net/publication/358117658.

[14] Dinega TM, Haile A, Beshir H. Improving onion productivity and producer income through nitrogen management. Adv Hort Sci. 2023;37(3):317–27. 10.36253/ahsc-13944.

[15] Aliyu U, Dikko AU, Magaji MD, Sing A. Nitrogen and Intra-row spacing effect on growth and yield of onion (*Allium Cepa* L). J Plant Sci. 2008;3(3):188–93. 10.3923/jps.2008.188.193.

[16] Khan IU, Jilani MS, Nadeem MA, Kiran M, Jilani TA, Saleem H. Impact of N-fertilization on onion bulb production of different genotypes through onion‐set. - Inter J Emerg Techn. 2021;12(2):161–154. https://www.researchgate.net/publication/354985343.

[17] Melkamu A, Minwyelet J. Optimum rates of NPS fertilizer application for the economically profitable production of potato varieties at Koga irrigation Scheme, Northwestern Ethiopia. Cogent Food Agric. 2018;4(1):1439663. 10.1080/23311932.2018.1439663.

[18] Regional M. Station, 2024.

[19] Asfaw Z, Eshetu D. Production and management of major vegetable crops in Ethiopia. Ethiop Inst Agricultural Res Addis Ababa Ethiopia. 2015;32–51. https://www.academia.edu/72044949/.

[20] MoA (Ministry of Agriculture). Plant varieties release, protection and seed quality control directorate. Crop variety register. Addis Ababa, Ethiopia. Issue Number 21. EIAR (Ethiopian Institute of Agricultural Research). Directory of released crop varieties and their recommended cultural practices. Addis Ababa, Ethiopia. 2018. http://hdl.handle.net/123456789/2404.

[21] Gomez AA, Gomez AA. Statistical procedures for agricultural research. 2nd edn. New York: John Wiley and Sons 680. 1984. http://pdf.usaid.gov/pdf_docs/pnaar208.pdf.

[22] Yeshiwas Y, Alemayehu M, Adgo E. Enhancing bulb yield through nitrogen fertilization and the use of hybrid onion (*Alluim Cepa* L.) varieties in Northwest Ethiopia. PLoS ONE. 2024;19(10):e0312394. 10.1371/journal.pone.0312394. 39446848 10.1371/journal.pone.0312394 PMC11500932

[23] Fikre M, Nikus O. Onion Seed Production Techniques: A Manual for extension agents and seed producers. FAO-CDMDP, Asella. Ethiopia. 2010. https://coin.fao.org/coin-static/cms/media/7/13029380384160.

[24] EIAR (Ethiopian Institute of Agricultural Research). Directory of released crop varieties and their recommended cultural practices. Ethiopia: Addis Ababa; 2012.

[25] Tsegaye B, Bizuayehu T, Woldemichael A, Mohammed A. Yield and yield components of onion (Allium Cepa L.) as affected by irrigation scheduling and nitrogen fertilization at Hawassa districts in Southern Ethiopia. J Med Biol Sci Res. 2016;2(2):15–20. https://www.researchgate.net/publication/355466583.

[26] Walle T, Dechassa N, Kebede WT. Yield and yield components of onion (Allium *Cepa* var. Cepa) cultivars as influenced by population density at Bir Sheleko, North-Western Ethiopia. Acad Res J Agri Sci Res. 2018;6(3):172–92. 10.14662/ARJASR2018.026.

[27] Alebachew M, Melkamu A, Biruk M. Optimal rate of nitrogen and intra row spacing for economical production of onion under irrigated farming system in Eastern Amhara Region, Ethiopia. Curr Res Agri Sci. 2019;6(2):83–94. 10.18488/journal.68.2019.62.83.94.

[28] Boukary H, Haougui A, Barage M, Adam T, Roumba A, et al. Evaluation for agro-morphology of onion varieties under ecotypes of Nigeria. Int J Biol Chem Sci. 2012;6:3098–106. http://www.scirp.org/reference/referencespapers?referenceid=1834207.

[29] Tekeste N, Dechassa N, Woldetsadik K, Dessalegne L, Takel A. Influence of nitrogen and phosphorus application on bulb yield and yield components of onion (*Allium Cepa* L). Open Agri J. 2018;12(1):194–206. 10.2174/1874331501812010194.

[30] Guesh T. Growth, Yield and Quality of Onion (Allium Cepa L.) As Influenced by Intra row Spacing and Nitrogen Fertilizer Levels at Central Zone of Tigray. Northern Ethiopia. Thesis, Haramaya University, Haramaya, Ethiopia. 2015;51–57. https://cgspace.cgiar.org/server/api/core/bitstreams/06140e5e-d4dd-4a48-8209-2e546ed43ed0/content.

[31] Birtukan A, Yohannes G, Asrat A. Bulb quality and storability of onion (Allium Cepa L.) as affected by varieties and intra row spacing in Antsokia Gemza, Ethiopia. Turkish J Agri - Food Sci Techn. 2020;8(3):580–86. 10.24925/turjaf.v8i3.580-586.2954.

[32] Rageb ME, Shaheen AM, Fatma A, Mahmoud S, Nadia M, et al. Effect of planting dates and NPK fertilizer levels on onion seeds production. Middle East J Agri Res. 2018;7(1):41–9. https://www.curresweb.com/mejar/mejar/2018/41-49.pdf?.

[33] Kumar RD, Ramchandra SS, Paikra DS. Effect of different nitrogen level on growth performance of onion under Poplar based agroforestry system. Int J Innovative Res Sci Eng Techno. 2018;7(10):2319–8753. 10.15680/IJIRSET.2018.0710010.

[34] SAS [Statistical Analysis Software], SAS/STAT. Version 9.2 computer software. Cary, NC: SAS Institute Inc., USA,; 2008.

[35] CIMMYT [Centro Internacional de Mejoramiento de Maíz Y Trigo]. From Agronomic Data to Farmers’ Recommendations: An Economic Training Manual, completely revised Edition, Mexico. 1988;79.

[36] Gebremedhn G, Yohanes G, Kiros A, Eyasu A, Weldegerima G, et al. Enhancing productivity and production of onion through use of improved varieties at North Western zone of Tigray, Ethiopia. Int J Envir Agri Biotech. 2018;3(3):0756–62. 10.22161/ijeab/3.3.6.

[37] Benti GB. Effect of nitrogen on growth, yield and shelf-life of onion (*Allium Cepa* L.) varieties in Fedis district. J Agri Nat Res Sci. 2017;4(1):1–22. www.journals.wsrpublishing.com/index.php/tjanrs/article/view/306/563.

[38] Khokhar KM. Environmental and cultural factors affecting flowering and bolting in onion – a review. Int J Vegetable Sci. 2019;25(3):267–78. 10.1080/19315260.2018.1488886.

[39] Boyhan GE, Schmidt NE, Purcell AE. Bolting in short-day onions: influence of cultivar and environment. Hort Sci. 2000;35(3):417.

[40] Villanova J, Roca M, Tomás R, Carrillo M. Breeding for bolting resistance in short-day onions for tropical and subtropical regions. Euphytica. 2021;217:142. 10.1007/s10681-021-02877-4.

[41] Weldemariam S, Gessesew K, Woldetsadik G, Wassu M. Growth parameters of onion (*Allium Cepa* L.) as affected by nitrogen fertilizer rates and Intra-row spacing under irrigation in Gode, South-East Ethiopia. Agri Forestry Fisheries. 2015;4(6):239–45. 10.11648/j.aff.20150406.11.

[42] Abbas MW, Bibi Y, Afzal M, Iqbal S, Mahmood K. Effect of different levels of nitrogen on growth, yield and quality of onion (Allium Cepa L.) under arid conditions. Pakistan J Bot. 2022;54(2):661–6. 10.30848/PJB2022-2(33).

[43] Rizk FA, El-Desouki MA, Mahmoud AA. Response of onion plants to different rates of nitrogen and potassium fertilization. J Soil Sci Agricultural Eng. 2020;11(12):729–34. 10.21608/jssae.2020.134331.

[44] Khan IU, Jilani MS, Nadeem MA, Kiran M, Jilani TA, Saleem H. Impact of nitrogen fertilization on onion bulb production of different genotypes through onion-Set. Int J Emerg Techno. 2021;12(2):161–70. https://www.researchgate.net/publication/354985343.

[45] Shoba TK, Rohini N, Arumugam T. Performance evaluation of aggregatum onion genotypes (Allium Cepa Var. aggregatum) for Yield, quality and resistance characters. Int J Curr Microbiol App Sci. 2017;6(6):634–42. 10.20546/ijcmas.2017.606.075.

[46] Mandefro T, Wondwosen T, Bizuayehu D. Effects of different nitrogen and sulfur fertilizer rates on growth, yield, quality and nutrient uptake of onion (*Allium Cepa* L.) at Shewa Robit, North Shewa, Ethiopia. Open Biotech J. 2021;15:59–67. 10.2174/1874070702115010059.

[47] Gebretsadik K, Dechassa N. Effect of nitrogen fertilizer rates and intra row spacing on bulb yield of onion (*Allium Cepa* L.) at Shire, Northern Ethiopia. Int J Sci Res. 2016;7(10):1769–73. 10.21275/ART20162419.

[48] Tiru T, Kebede W, Wondimu B. Shallot Yield, quality and Shelf-life as affected by nitrogen. Int J Vegetable Sci. 2015;21(5):454–66. 10.1080/19315260.2014.895790.

[49] Al-Fraihat AH. Effect of different nitrogen and sulphur fertilizer levels on growth, yield and quality of onion (*Allium Cepa* L). Jordan J Agri Sci. 2009;5(2):155–66. https://www.researchgate.net/publication/306032586.

[50] Morsy MG, Marey RA, Karam SS, Abo-Dahab AM. Productivity and storability of onion as influenced by the different levels of NPK fertilization. J Agricultural Res Kafer El-Sheikh Univ. 2012;38(1):171–86.

[51] Brady NC. The Nature and Properties of Soils, 9th Edn. New Delhi. 1985.

